# Health Consequences of Typhoon Haiyan in the Eastern Visayas Region Using a Syndromic Surveillance Database

**DOI:** 10.1371/currents.dis.4a3d3b4474847b2599aa5c5eefe3a621

**Published:** 2017-02-06

**Authors:** Miguel Antonio Salazar, Ronald Law, Arturo Pesigan, Volker Winkler

**Affiliations:** Institute of Public Health, Ruprecht Karls Universitat Heidelberg, Heidelberg, Germany; Faculty of Management and Development Studies, University of the Philippines Open University, Los Baños, Laguna, Philippines; Health Emergency Management Bureau, Department of Health, Manila, Philippines; Health Emergencies Programme, World Health Organization Regional Office for South-East Asia, New Delhi, India; Institute of Public Health, Ruprecht Karls Universitat Heidelberg, Heidelberg, Germany

## Abstract

Introduction: Typhoon Haiyan was the strongest storm recorded in Philippine history. Surveillance in Post Extreme Emergencies and Disasters (SPEED) was activated during the typhoon response. This study analyzes the health impact of different diseases during different timeframes post-disaster during Typhoon Haiyan in 2013 using a syndromic surveillance database.

Methods: SPEED reports medical consultations based on 21 syndromes covering a range of conditions from three syndrome groups: communicable diseases, injuries, and non-communicable diseases (NCDs). We analyzed consultation rates for 150 days post-disaster by syndrome, syndrome group, time period, and health facility type for adults as well as for children under the age of five.

Results: Communicable diseases had the highest consultation rates followed by similar rates for both injuries and NCDs. While communicable diseases were the predominant syndrome group for children, wounds and hypertension were common syndromes observed in adults. Village health centers had the most consultations amongst health facilities, but also showed the highest variability.

Discussion: Children were more vulnerable to communicable diseases compared to adults. Community health centers showing consistently high consultation rates point out a need for their prioritization. The predominance of primary care conditions requires disaster managers to focus on basic health care and public health measures in community health centers that target the young, elderly and impoverished appropriate to the time period.

## Introduction

Typhoon Haiyan was a category five typhoon, which made landfall in the Philippines on November 8, 2013. It made 6 landfalls in the Visayas and Palawan and caused considerable damage to these regions. It affected around 16 million people and killed more than six thousand.^1^^,^ 2 It is considered a sudden onset disaster with prolonged health consequences.^1^^,^ 3

The Health Emergency Management Bureau (HEMB) of the Philippine Department of Health (DOH) activated the Surveillance in Post Extreme Emergencies and Disasters (SPEED) during the response to Typhoon Haiyan. SPEED is a syndromic surveillance system for health facilities that monitors 21 syndromes. Reports can be transmitted manually, via mobile or the Internet. SPEED runs in parallel with the regular epidemiologic reporting system of the DOH. SPEED’s database records consultation rates for 21 syndromes. It enables early detection of communicable diseases, non-communicable diseases, and injuries, monitors syndrome trends, and guides the DOH’s disaster response.^4^^,^ 5

Typhoon Haiyan was a distinct event that disrupted health systems of various regions. Ensuing storm surge caused most of the damage to the population and infrastructure, such as water, electricity, and telecommunication. Typhoon Haiyan dealt a severe blow to the public health system with damages to the tune of 26 million US dollars (USD). The damage to the health infrastructure including 42 hospitals, 95 community health centers and 427 village health centers alone accounted for 19.2 million USD.^1^^,^ 6

Typhoons in general can pose a risk for the outbreak of infectious diseases. Such outbreaks are usually endemic to the affected area and not unfamiliar to the local medical personnel. Expected diseases include watery diarrhea, acute respiratory infections, measles, malaria and dengue. The environmental conditions of overcrowding, increased vector spread, lack of clean drinking water, and hygiene facilities contribute to these conditions.^7^^,^ 8 ^,^ 9 ^,^ 10 Thus humanitarian organizations prioritize these infectious diseases in their disaster response.^11^

Although the DOH and the World Health Organization (WHO) issued publications on Typhoon Haiyan none of these studies include a detailed account of the disaster’s health impact.^1^^,^ 2 ^,^ 6 In 2015 Martinez et al. analyzed SPEED and conducted focused group discussions to show the health impact of non-communicable diseases during Haiyan.^12^ In 2016 Salazar et al. used the SPEED database to, analyze the health impact of three disasters from 2013 including Typhoon Haiyan. This analysis, however, did not give detailed information on individual syndromes, health facilities, and children.^10^ The main objective of this study is to analyze the health impact of communicable diseases, injuries, and non-communicable diseases (NCD) during Typhoon Haiyan using the SPEED database including descriptions of individual syndromes and the use of health facilities by different age groups during disaster response and recovery.

## Methods

This is a descriptive study of an existing database of the Health Emergency Management Bureau. SPEED uses aggregated data from health facilities in disaster affected areas. This study analyzes SPEED data from health facilities in the Eastern Visayas region, one of the four regions affected by Typhoon Haiyan, from November 8, 2013 to March 28, 2014.^6^

The database comprises reports that detail the number of consultations per day by health facility for two age groups (<5 vs. ≥5 years of age).^4^ This study categorizes all syndromes into three groups: communicable diseases, injuries, and NCDs similar to the study by Salazar et al. from 2016.^10^ Communicable diseases include the following syndromes: acute bloody diarrhea, acute flaccid paralysis, acute hemorrhagic fever, acute jaundice syndrome, acute respiratory infection, acute watery diarrhea, animal bites, conjunctivitis, fever, fever with other symptoms, skin disease, suspected leptospirosis, suspected measles, suspected meningitis, and tetanus. Fractures and wounds, including bruises and burns are considered syndromes for injuries. The following are syndromes for NCD’s: acute asthmatic attack, acute malnutrition, high blood pressure, and known diabetes mellitus.^4^^,^ 10

To calculate consultation rates per day according to health facility, age group as well as all ages combined, we divided the respective number of consultations by the corresponding population of the catchment area of each health facility. The catchment area was estimated according to the type of health facility i.e. village health centers, community health centers, hospitals, evacuation centers, mobile clinics, foreign medical team clinics, and foreign medical team hospitals. For village health centers and evacuation centers the village population was defined as the catchment area. For community health centers and hospitals the municipality or city population was used. Less observed health facility types (mobile clinics 1%, foreign medical team clinics 2%, and foreign medical team hospitals 0.4%) were merged with the best fitting group as follows: mobile clinics and foreign medical team clinics with village health centers and foreign medical team hospitals with hospitals. The population data used is based on the 2010 census from the Philippine Statistics Office.^13^^,^ 14

Mean consultation rates per day with 95% confidence intervals and t-tests comparing age groups and time periods were calculated using Stata Version 13.^15^^,^ 16 Scatter plots and splines were used to visualize the trend in syndrome morbidity. Splines are based on regressions, joining points to fit a particular shape.^17^ Consultation rates were presented per 10,000 individuals per day as this number is routinely used in the literature for emergency health kits.^10^^,^ 18 Additionally, rates within two months (≤60 days) were compared to rates after two months (>60 days) as this was identified to separate response and recovery.^10^

As this is a descriptive study of an existing database using aggregated data, the Institutional Review Board of Heidelberg University deemed the study exempt from full review. Prior to the start of the study, the SPEED database for Typhoon Haiyan was requested from the director of HEMB. HEMB did an initial validation of the Haiyan data.^10^ All SPEED database reports were handled with confidentiality by the authors of this study. SPEED consists of aggregated data from health facilities, which does not include individual patient information, thus also ensuring the confidential and anonymous nature of the database.

## Results

As part of disaster disease surveillance conducted by HEMB and DOH, there were 3,425 SPEED reports for Typhoon Haiyan in the Eastern Visayas region within 150 days post-disaster. For mean rates, communicable diseases had overall the highest rates, 47.3 per 10,000 individuals, followed by similar rates for both injuries (5.9) and NCDs (6.0). Looking at consultation rates over time, the representative syndromes for communicable diseases, injuries, and NCDs are acute respiratory infections, wounds, and high blood pressure respectively. These syndromes were selected because they had the highest rates among all syndrome groups (see**Table 1**). The spline and scatter plot shows a peak in consultation rates around day 20 to 25 for all three syndromes. Thereafter, rates decrease and stabilize around day 50 post-disaster. Acute respiratory infections showed a much more prominent increase in consultation rates compared to wounds and high blood pressure (see **Figure 1**).

**Table:** **Table 1:** Syndrome rates per 10,000 individuals separated by time post-disaster and by age

Syndrome	Total	≤ 2 months	> 2 months	Difference between ≤ 2 months and > 2 months	< 5 years of age	≥ 5 years of age	Difference between < 5 years and ≥ 5 years
Communicable diseases							
Acute respiratory infection	36.0	64.0	11.1	52.9 (p<0.01)	112.4	25.6	86.9 (p<0.01)
Skin disease	4.5	8.2	1.2	7.0 (p<0.01)	13.8	3.2	10.5 (p<0.01)
Acute watery diarrhea	2.5	4.6	0.6	4.0 (p<0.01)	11.4	1.3	10.0 (p<0.01)
Fever	2.3	4.1	0.7	3.4 (p<0.01)	8.4	1.4	6.9 (p<0.01)
Fever with other symptoms	0.8	1.3	0.3	1.0 (p<0.01)	1.4	0.7	0.7 (p=0.06)
Animal bites	0.5	0.8	0.2	0.6 (p=0.03)	1.1	0.4	0.8 (p=0.11)
Conjunctivitis	0.3	0.5	0.1	0.4 (p<0.01)	0.5	0.3	0.2 (p=0.19)
Suspected leptospirosis	0.1	0.3	<0.1	0.3 (p=0.01)	0.2	0.1	0.1 (p=0.52)
Acute bloody diarrhea	0.1	0.2	<0.1	0.2 (p<0.01)	0.3	0.1	0.3 (p=0.01)
Suspected meningitis	0.1	0.2	<0.1	0.2 (p=0.24)	0.1	0.1	<0.1 (p=0.78)
Suspected measles	0.1	0.1	0.1	<0.1 (p=0.47)	0.5	<0.1	0.4 (p=0.07)
Acute hemorrhagic fever	<0.1	<0.1	<0.1	<0.1 (p=0.34)	0.1	<0.1	<0.1 (p=0.28)
Acute jaundice syndrome	<0.1	<0.1	<0.1	<0.1 (p=0.04)	<0.1	<0.1	<0.1 (p=0.03)
Tetanus	<0.1	<0.1	<0.1	<0.1 (p=0.12)	<0.1	<0.1	<0.1 (p=0.18)
Acute flaccid paralysis	<0.1	<0.1	<0.1	<0.1 (p=0.02)	<0.1	<0.1	<0.1 (p=0.03)
Communicable disease total	47.3	84.5	14.2	70.3 (p<0.01)	150.2	33.3	116.9 (p<0.01)
Injuries							
Open wounds & bruises/burns	5.8	10.7	1.4	9.3 (p<0.01)	6.2	5.7	0.4 (p=0.74)
Fractures	0.1	0.2	<0.1	0.2 (p=0.01)	0.3	0.1	0.2 (p=0.25)
Injury total	5.9	10.9	1.4	9.5 (p<0.01)	6.4	5.8	0.6 (p=0.65)
Non-communicable diseases							
High blood pressure	4.7	8.0	1.8	6.2 (p<0.01)	0.1	5.4	5.3 (p<0.01)
Acute asthmatic attack	0.9	1.6	0.3	1.3 (p<0.01)	2.0	0.8	1.2 (p<0.01)
Known diabetes mellitus	0.4	0.7	0.1	0.6 (p<0.01)	<0.1	0.4	0.4 (p<0.01)
Acute malnutrition	<0.1	0.1	<0.1	0.1 (p=0.04)	0.2	<0.1	0.2 (p=0.22)
NCD Total	6.0	10.3	2.3	8.1 (p<0.01)	2.2	6.6	4.3 (p<0.01)

**Figure:**
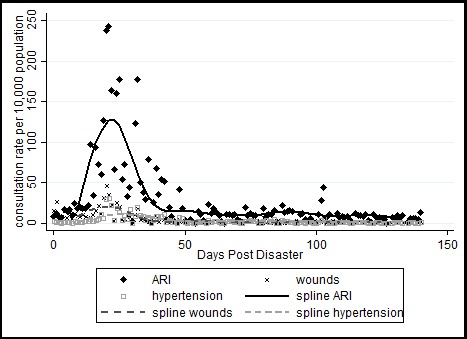
**Fig. 1:** Consultation rates per 10,000 individuals for acute respiratory infections, wounds, and hypertension.

A similar number of reports was observed within two months (n=1,614) and after two months (n=1,811) post-disaster. Within two months post-disaster, acute respiratory infections also accounted for the highest consultation rates with 64.0 per 10,000 individuals. Wounds and hypertension had consultation rates of 10.7 and 8.0 per 10,000 individuals respectively. After two months post-disaster the rates for all three syndromes were significantly lower: acute respiratory infections (11.1), wounds (1.4), and hypertension (1.8).

Rates for the whole 150-day period decreased in the following order: acute respiratory infections, open wounds, high blood pressure, skin disease, acute watery diarrhea, and fever. Within two months post-disaster, eight syndromes showed consultation rates higher than 1 per 10,000 individuals compared to six for the whole period. The additional two syndromes were acute asthmatic attack and fever with other symptoms. After two months post-disaster, only four syndromes were above 1 consultation per 10,000 individuals per day. These were acute respiratory infections, open wounds, high blood pressure, and skin disease. Comparing consultation rates for these two periods all syndromes show higher consultation rates in the first two months post-disaster. Only four syndromes displayed differences with p values greater than 0.05; these were meningitis, suspected measles, acute hemorrhagic fever, and tetanus. The top eight syndromes: acute respiratory infections, open wounds, hypertension, skin disease, watery diarrhea, fever, asthma, and fever with other symptoms showed differences greater than 1 consultation per 10,000 individuals and p values less than 0.01 (see **Table 1**).

The top eight syndromes showed differences when analyzed according to age group. Acute respiratory infections, skin disease, acute watery diarrhea, and fever showed the highest consultation rates for children under the age of five. Acute respiratory infections had the highest consultation rates for adults followed by wounds, hypertension, and skin disease. Communicable diseases had significantly higher rates in under-five-year old children specifically for acute respiratory infections, skin disease, acute watery diarrhea, and fever. Injuries had similar rates for both age groups. While NCDs in form of hypertension and diabetes were more common for adults, asthma was more common for children under the age of five (see **Table 1**).

When grouped according to health facility type, most records in SPEED were contributed by community health centers and hospitals with 54% and 30% respectively. Village health centers had the highest consultation rates among the four health facility types and also had the highest variability across time seen in its 95% confidence interval and difference in the mean rates (see **Table 2**).

**Table:** **Table 2:** Consultations per 10,000 individuals per day per health facility

Health Facility Type	# of SPEED reports	Mean with 95% confidence interval
Community Health Center	1850	25.0 (20.3-29.7)
Evacuation Center	39	19.5 (0-43.9)
Hospital	1039	7.0 (6.5-7.6)
Village Health Center	497	299.0 (247.1-350.9)

## Discussion

This study provides a detailed analysis of the conditions seen in health facilities in the aftermath of Typhoon Haiyan. It illustrates that communicable diseases were the most common syndrome group, and that acute respiratory infection was the most common of the 21 syndromes. Under-five-year old children displayed higher rates for communicable diseases, similar rates for injuries, and lower rates for NCDs in comparison with adults. The highest consultation rates recorded came from village health centers followed by community health centers.

Syndromes which were consistent in their consultation rates throughout the whole period, such as meningitis, suspected measles, acute hemorrhagic fever, and tetanus all require referral to tertiary care.^19^ These syndromes showed rates of less than 0.1 for the whole 150-day post-disaster period, indicating nevertheless a potential risk these syndromes pose during response and recovery periods. The most common syndromes recorded in the SPEED database represented primary care conditions. According to Connelly et al. in 2004, acute respiratory infections and diarrhea, syndromes which were also observed in this study, account for the majority of morbidities in emergencies. Acute respiratory infections and diarrhea are attributed to over-crowding in shelters and lack of quality water services respectively.^20^

Communicable diseases were more prominent in children under five manifesting as acute respiratory infections, skin disease, acute water diarrhea, and fever. A study concluded in the aftermath of Hurricane Katrina in the United States in 2005, showed that pediatric consultations included respiratory conditions, skin ailments, and under-nutrition. Respiratory conditions reported were acute respiratory infections and allergic rhinitis while skin conditions included bacterial and fungal infections.^21^ Furthermore, a systematic review from 2013 showed that the most common pediatric themes found in the literature for emergencies and disasters included infectious conditions, vaccine-treatable diseases, hygiene-related ailments, and wounds.^22^

As expected NCDs were more visible in adults; hypertension had the third highest rate. Moreover, hypertension and diabetes displayed a significant difference in rates within and after two months post-disaster. Another study on Hurricane Katrina found that 40% of those seeking consultations came because of chronic conditions such as hypertension. 20% came in order to get medication for pre-existing chronic conditions.^23^ This may be due to an increase in demand for chronic disease treatment because of health system disruption.^24^ In the case of Typhoon Haiyan, first-line health providers saw that patients with NCDs experienced a lack of supply of medications and basic necessities such as food and clothing.^12^ The need for basic provisions for survival may have pushed these patients to seek consult for pre-existing conditions thus causing an increase in the consultation rates within the first two months post-disaster.

Haiyan’s mortality profile and magnitude causing disaster response delay contributed to the conditions observed in SPEED. Most storm surge mortalities are due to drowning.^25^ This is also true for Typhoon Haiyan. In a case control study after the typhoon, which looked into the risk factors associated to mortality, the deaths observed were all due to drowning.^26^ Since the disaster had a high death toll (6,300 people were killed),^1^ consultation rates for fractures were low. Open wounds, the more benign syndrome, had the second highest syndrome rate. Moreover, the initial responders were also victims since their health facilities and homes had been destroyed by the typhoon. It took the local responders from neighboring regions two days before they reached the hardest hit areas.^1^ Furthermore, foreign medical teams needed a minimum of three days after arriving in the country to be operational. The peak of functional number of foreign medical teams was only achieved 22 days post-disaster.^3^ The delay in the response to Typhoon Haiyan may have shifted the profile of the diseases seen in SPEED to more primary care conditions.

The top syndromes highlighted in this study can be addressed with proper diagnosis, appropriate medications, and wound cleaning & dressing.^19^ Due to their permanent vulnerability, poor populations and people with chronic conditions should be considered for targeted interventions during response and recovery phases of disasters.^24^ In general, communicable diseases, injuries, and NCDs can be addressed by providing continuous basic health services, water, sanitation and hygiene, nutrition, public health surveillance, disease control, and proper shelter to vulnerable groups namely poor, young, and elderly patients.^10^^,^ 11 ^,^ 20 ^,^ 27

For most syndromes regardless of need for referral, rates within two months were significantly higher than those after two months. In the case of Typhoon Haiyan, the Interagency Standing Committee downgraded its emergency response level 93 days or three months post-disaster. This was in contrast to the national government decision to move from disaster relief to recovery seven months post-disaster.^28^ Compared to these bureaucratic decisions by both national and international agencies, SPEED provides an evidence-based junction between disaster response and recovery seen in the trends of consultations post-disaster. The sustained decrease in consultations around day 50 can be constituted as the demarcation between disaster response and recovery based on disease surveillance trends.^10^

We additionally calculated median values to take into account outliers in the data set, but interpretation of the results remained similar to mean rates. Due to the lower number of SPEED reports for evacuation centers and village health centers the mean rates had wider confidence intervals compared to community health centers and hospitals. This high variability prompts further research in health facility utilization during disasters.

Typhoon Haiyan was a mega-disaster with extensive disruption of the health system. The Eastern Visayas region had 22 hospitals, 53 community health centers, and 113 village health centers damaged comprising 52%, 56%, and 26% respectively of each of the damaged health facility types across all four regions.^2^ Since SPEED received most data from community health centers and hospitals we suppose that these health facility types were the ones to be still operational. Another explanation might be that disaster managers and health ministry officials prioritized these central health facilities as the main reporting centers due to limited capability of logistics and transportation to get to the village health centers further away. This prioritization of community health centers and hospitals was reflected in the publications of the WHO Philippine Country Office and the DOH on community health centers and hospitals affected by Typhoon Haiyan. Community health centers were described as the focal point of primary health care. The local health executives govern the management of village health centers in their catchment areas through community health centers. Moreover, these act as feeders to nearby hospitals for tertiary care.^2^^,^ 6

This study demonstrates the use of SPEED for planning response and recovery activities. In the prioritization of health facilities to be reconstructed or rehabilitated, we propose that the health facilities showing significantly higher rates of consultations, namely community health centers, should be prioritized immediately. Logistics for required medications and supplies for different age groups should be planned according to their percentage in the local population. Planning for medicines, supplies, and equipment as well as training on protocols for prominent diseases of children and aging populations should be emphasized. These populations are also less mobile thus access to health services, both financial and geographic, should be considered. As children and elderly are more vulnerable to environmental factors such as cold and wet weather,^29^ priority should be placed on shelter for these populations.

The rates for Typhoon Haiyan should be the benchmark for future planning purposes. Typhoon Haiyan was a category five typhoon and the strongest typhoon ever recorded in Philippine history.^2^ Thus, for now, it is the best template for preparing for the worst possible scenario.

Being a syndromic surveillance system, SPEED has some inherent limitations with regard to the generalizability of the results of this study. The findings of the study refer to health facility data in the Eastern Visayas region and cannot be interpreted as being representative of the whole population affected. Moreover, the population data used to calculate the consultation rates did not take into account the actual population immediately before or after Typhoon Haiyan. Out-migration from the affected areas has not been taken into account either. With regard to SPEED itself, it has to be noted that in some of the affected areas, the system was not operational until one week post-disaster.^5^ As the syndromes are not that specific and health facilities were not able to report daily, the results should be read as rough estimates rather than exact values. Thus disaster managers still should also consider their extensive experience and integrate it with the findings of our study.

## Conclusions

The predominance of primary care conditions seen in the study may be used for planning for future disasters and signals disaster managers to focus on basic health care and public health measures. The trends observed for consultation rates across time may be used as guides for disaster response and recovery. Interventions targeting young, elderly, and impoverished populations are recommended. Community health centers should be prioritized in recovery and rehabilitation efforts.

## Competing Interests

The authors have declared that no competing interests exist.

## Corresponding Author

Miguel Antonio Salazar: miguel.salazar@upou.edu.ph

## Ethics

This study uses the 2013 data from Surveillance in Post Extreme Emergencies and Disasters (SPEED), an existing database using aggregated health facility data of the Health Emergency Management Bureau of the Philippine Department of Health. The authors of the study received the SPEED reports as aggregated data and did not have access to individual patient information.

## Data Availability

The data from this study are owned by the Health Emergency Management Bureau of the Department of Health of the Republic of the Philippines. As these are government-owned data, their release is subject to the approval of the Director of the Health Emergency Management Bureau. Requests for this data set may be sent to Director Gloria J. Balboa, MD, MPH, MHA of the Health Emergency Management Bureau via hembdiroffice@gmail.com, or through the following address: Health Emergency Management Bureau, Department of Health, San Lazaro Compound, Tayuman, Sta. Cruz, Manila, 1003 Philippines.

## Authors' Contribution

The authors of this manuscript were involved in the conception and design of the study or have contributed to the acquisition, analysis, or interpretation of the data of the study. They were part in its critical revision. They have agreed to be accountable for all aspects of the work.

MAS wrote the manuscript, conceptualized the study, interpreted the results, and collated the responses and comments from other authors. AP took part in the conception of the study and analyzing the results. RL was vital in acquiring data and analyzing the results. AP and RL contributed to the revision of the manuscript. VW contributed to the writing of the manuscript, the conceptualization of the study and the analysis of the results.
